# A Broad Requirement for TLS Polymerases η and κ, and Interacting Sumoylation and Nuclear Pore Proteins, in Lesion Bypass during *C. elegans* Embryogenesis

**DOI:** 10.1371/journal.pgen.1002800

**Published:** 2012-06-28

**Authors:** Sophie F. Roerink, Wouter Koole, L. Carine Stapel, Ron J. Romeijn, Marcel Tijsterman

**Affiliations:** Department of Toxicogenetics, Leiden University Medical Center, Leiden, The Netherlands; Duke University, United States of America

## Abstract

Translesion synthesis (TLS) polymerases are specialized DNA polymerases capable of inserting nucleotides opposite DNA lesions that escape removal by dedicated DNA repair pathways. TLS polymerases allow cells to complete DNA replication in the presence of damage, thereby preventing checkpoint activation, genome instability, and cell death. Here, we characterize functional knockouts for *polh-1* and *polk-1*, encoding the *Caenorhabditis elegans* homologs of the Y-family TLS polymerases η and κ. POLH-1 acts at many different DNA lesions as it protects cells against a wide range of DNA damaging agents, including UV, γ-irradiation, cisplatin, and methyl methane sulphonate (MMS). POLK-1 acts specifically but redundantly with POLH-1 in protection against methylation damage. Importantly, both polymerases play a prominent role early in embryonic development to allow fast replication of damaged genomes. Contrary to observations in mammalian cells, we show that neither POLH-1 nor POLK-1 is required for homologous recombination (HR) repair of DNA double-strand breaks. A genome-wide RNAi screen for genes that protect the *C. elegans* genome against MMS–induced DNA damage identified novel components in DNA damage bypass in the early embryo. Our data suggest SUMO-mediated regulation of both POLH-1 and POLK-1, and point towards a previously unrecognized role of the nuclear pore in regulating TLS.

## Author Summary

Unrepaired DNA damage on the template strand poses a problem for the progression of the replication fork. Specialized translesion synthesis (TLS) polymerases are capable of bypassing DNA lesions without repairing them. Here, we use the nematode *C. elegans*, to show that there is modulation of the choice between repair and bypass during development. We show that during gametogenesis and later development repair dominates, while there is a short phase during embryonic development where resistance to damage depends heavily on TLS polymerases. The rapid divisions at this stage do not allow for delay in which repair processes can occur. Furthermore, we identify new factors that may play a role in the regulation of TLS during early embryogenesis.

## Introduction

DNA damaging agents from both endogenous and exogenous sources can induce replication-blocking DNA lesions that threaten cell cycle progression and, consequently, cell viability. To remove these DNA lesions cells are equipped with various specialized repair mechanisms [1], including nucleotide excision repair (NER) that deals with helix-distorting obstructions [2]. However, during embryogenesis, which entails phases of rapid cell division, only a limited time window is available for repair processes [3]. Consequently, unrepaired DNA damage may delay replication and cell cycle progression. In the nematode *Caenorhabditis elegans* a delay in replication is detrimental for the developmental program; timing of cell division is fixed and strictly regulated by the homologues of the checkpoint genes CHK1 and ATR [4]. Indeed, replication stress caused by depletion of nucleotide pools causes fatal errors in the correct timing of the first asynchronous divisions [5]. However, early embryos of *C. elegans* appear to be remarkably resistant to DNA damaging agents, suggesting an efficient way to prevent the induction of replication stress by DNA damage [6].

To be able to deal with replication obstructions, organisms evolved ways that allow bypass of the damaged template, thus ensuring continuity of the replication process [7]. Specialized TLS polymerases are capable of direct bypass of DNA lesions in an error free or error prone fashion, depending on their affinities for the specific lesion site. In eukaryotes, TLS is mediated by the DNA polymerases of the Y-family: Polη, Polκ, Polι and Rev1, and the B-family member Polζ. All members of the Y-family polymerases lack proofreading activity and share a conserved active site, which is different from the high-fidelity polymerases in its open and less sterically constrained structure. It allows for accommodation of a DNA lesion, but is also the basis for reduced fidelity [8]. The functional specificities of TLS polymerases are due to minor differences in the structural features of the active site.

The *C. elegans* genome encodes several Y-family TLS proteins, including POLH-1 and POLK-1, homologs of mammalian Polη and Polκ, respectively. Purified Polη of yeast and vertebrates is capable of replicating across a wide variety of DNA damages, including UV-induced cyclobutane pyrimidine dimers (CPDs), 7,8-dihydro-8-oxoguanine, *O* ^6^-methylguanine, thymine glycol, cisplatin-induced intrastrand crosslinks, acetylaminofluorene-adducted guanine and benzo[*a*]pyrene-*N* ^2^-guanine [9]. In humans, defective Polη has clinical implications: Polη is the product of the gene mutated in Xeroderma Pigmentosum complementation group “Variant” (XPV), a syndrome that is associated with a high predisposition towards developing skin cancers [10]. In addition to a role in damage bypass some studies have suggested a role for Polη in homologous recombination, as the polymerase responsible for extension of the invading strand in the D-loop recombination intermediate [11], [12]. Recently, it was reported that Polη plays a prominent role in early stages of nematode embryogenesis in *C. elegans* [6], [13]. Polκ displays structural similarity to Polη but is considered to be the most evolutionarily conserved member of the Y-family showing homology to prokaryotic DinB [8], [9]. Its substrate specificity *in vitro* is limited, although Polκ is an efficient extender of mispaired primer termini and some guanyl adducted lesion sites [14], [15]. Furthermore, Polκ has been suggested as one of the gap-filling polymerases in NER, explaining a moderate sensitivity of Polκ-deficient mammalian cells to UV [16], [17].

Here, we characterize the involvement of Polη and Polκ in various aspects of genome protection during animal development, using the model organism *C. elegans*. The advantages of this animal model are its spatial and temporal organization of gametogenesis and its rapid growth properties that allow monitoring DNA repair or lesion bypass during different developmental stages. We found that POLH-1 is involved in protection against a surprisingly wide range of DNA lesions, whereas the substrate specificity of POLK-1 is much more restricted. Both proteins can act redundantly on some lesions, since double mutants were extremely sensitive to the alkylating agent MMS, whereas both single mutants displayed profoundly less sensitivity to this carcinogen. In spite of their error proneness, POLH-1 and POLK-1 appear to be highly important in protection against DNA damage during embryonic development, while their role in later somatic development is limited. Finally, we used genome-wide RNAi to screen for factors that have a similar sensitivity profile leading to the identification of new factors that may play a role in the regulation of TLS.

## Results

### Isolation of *C. elegans* mutants for *polh-1* and *polk-1*

**Figure 1:**
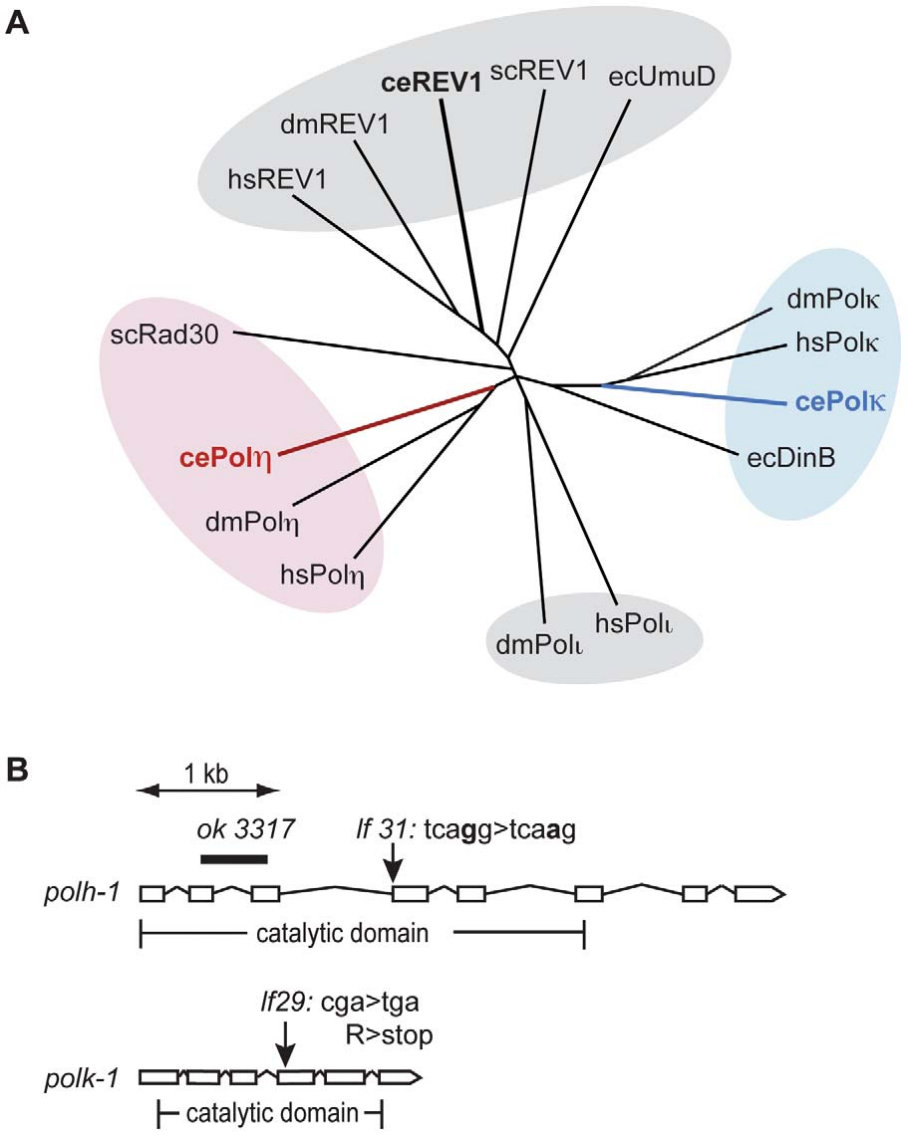
Y-family polymerases POLH-1 and POLK-1 of *C. elegans*. (A) Phylogenetic tree displaying Y-family polymerases from *C. elegans, S. cerevisiae, D. melanogaster, E. coli* and *H. sapiens*. Respectively red and blue branches show *C. elegans* POLH-1 (Polη) and POLK-1 (Polκ). (B) Gene structure of the *C. elegans polh-1* and *polk-1* genes and the molecular nature of the alleles used in this study.

To study the function of Y-family TLS polymerases in the DNA damage response at different stages of animal development, we set out to isolate mutants for the *C. elegans* homologs of the Polη and Polκ genes. Figure 1A illustrates a phylogenetic tree of the Y-family polymerase members from several species including *C. elegans*. The *C. elegans* genome encodes Polη, Polκ and Rev-1, but not Polι.

Full alignment of the *C. elegans polh-1* and *polk-1* gene products with their mammalian and yeast homologs reveals their well-conserved catalytic core (Figure S1). In addition, POLH-1 contains a C-terminal PIP box motif, which is essential for interaction with PCNA, and more recently, has also been shown to target the protein for degradation [13], [18]. The remaining part of the C-terminus is evolutionary less conserved. Human Polη/yeast Rad30 and human Polκ contain ubiquitin binding zinc finger (UBZ) domains, mediating their interaction with PCNA [19]. A UBZ domain was found in *C. elegans* POLK-1 but not in *C. elegans* POLH-1 (Figure S1). Furthermore, *C. elegans* and yeast Polη and Polκ lack previously identified mammalian motifs that mediate an interaction with the deoxycytidyl transferase Rev1 [20], [21].

Using a targeted mutagenesis approach [22] we isolated mutants for *polh-1* and *polk-1* (Figure 1B). *polh-1 (lf31)* has a single nucleotide substitution in the splice acceptor site of the fourth exon of the *polh-1* gene; *polk-1 (lf29)* contains a premature stop in the fourth exon and encodes a severely truncated version of POLK-1, missing at least part of the catalytic domain. In the course of this study we obtained another *polh-1* mutant from the Gene Knockout Consortium. This allele *(ok3317)* carries a deletion in *polh-1* that results in a fusion of upstream sequences of exon 2 to downstream sequences of exon 3, removing 549 coding nucleotides (Figure 1B). These mutant strains were backcrossed to remove background mutations that resulted from the mutagenic treatment. No obvious abnormal phenotypes were observed for the mutant strains. Neither the number of progeny, embryonic survival rate nor post-embryonic development was affected by the absence of POLH-1 or POLK-1. However, double mutants of *polh-1 (ok3317); polk-1 (lf29)* and of *polh-1 (lf31)*; *polk-1 (lf29)* show a minor but significant reduction in both brood size and embryonic survival (up to five percent of the progeny died, data not shown), suggesting some level of functional redundancy in promoting fecundity.

### *C. elegans* POLH-1 in protection against UV and cisplatin

**Figure 2:**
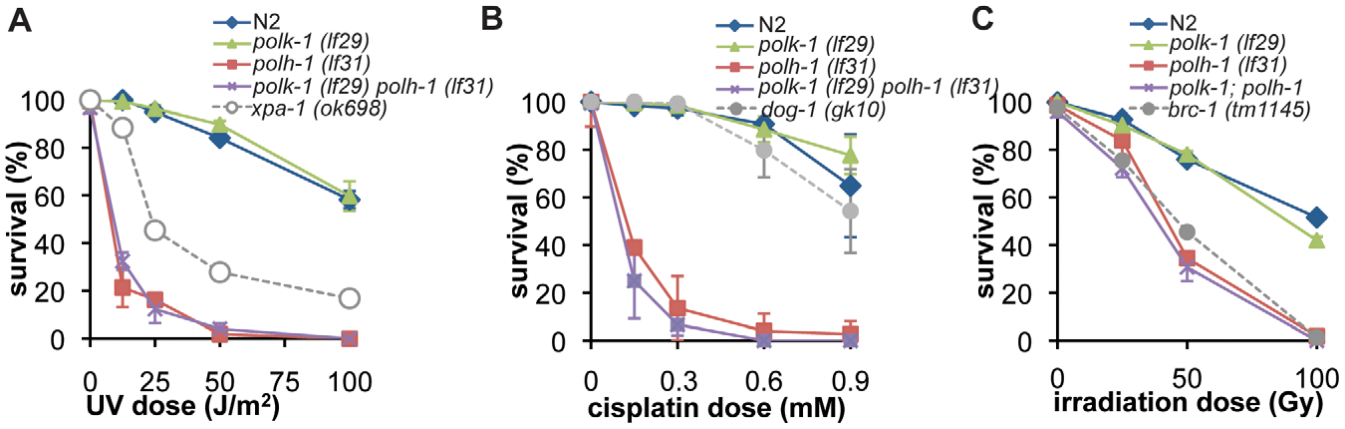
Germline sensitivity of *polh-1* and *polk-1* mutants to different sources of DNA damage. (A) Sensitivity to UV-irradiation. (B) Sensitivity to γ-irradiation. (C) Sensitivity to cisplatin. Adults were exposed to DNA damaging treatments and survival was quantified by counting dead embryos versus living progeny in the next generation. Each line represents the mean of minimal three independent experiments. Error bars denote the s.e.m.

Because UV-induced CPDs are excellent substrates for Polη-mediated TLS in yeast and mammals [23], [24], we tested the sensitivity of *polh-1* mutant animals to UV light by irradiating young adults and scoring progeny survival (Figure 2A and Figure S2A). In contrast to Polη-defective yeast and mammalian cells, that display only a mild hypersensitivity to UV [25], [26], POLH-1 deficiency leads to extreme sensitivity to UV irradiation. Both *polh-1(lf31)* and *polh-1(ok3317)* mutants are more sensitive to UV than animals carrying mutations in *xpa-1*, the worm homolog of NER gene XPA, which is essential for repair of UV damage [26], [27]. NER contributes to UV survival also in *polh-1* compromised conditions as animals defective in both *xpa-1* and *polh-1* are more sensitive than either of the single mutants. (Figure S2B). In line with mammalian data, we observed that the protective role of POLH-1 is not restricted to UV-induced damage. *polh-1* worms are severely sensitized to cisplatin treatment (Figure 2B and Figure S2C). This sensitivity was even more pronounced than for *dog-1* mutant animals which are defective in the homolog of the Fanconi Anemia gene FANCJ, involved in crosslink repair [28].

### POLH-1 and XPA-1 protect against γ-irradiation–induced damage in the *C. elegans* germline

Vertebrate Polη has been implicated as the polymerase responsible for extension of HR intermediates [12], [29]. Since HR is the predominant repair pathway in *C. elegans* for γ-irradiation-induced breaks in germ cells [30], [31], we exposed L4 animals to γ-irradiation and scored survival of the progeny (Figure 2C and Figure S2D). We found that the sensitivity of *polh-1 (lf31)* and *polh-1 (ok3317)* mutants to irradiation was comparable to the sensitivity of animals that carry a mutation in *brc-1*, the worm homolog of the HR gene BRCA1 (Figure 2C). Worms defective for both *polh-1* and *brc-1* were more sensitive to γ-irradiation than either of the single mutants (Figure S2E–S2F), suggesting a *brc-1*-independent role for POLH-1 in protection against γ-irradiation. This conclusion is strengthened by data showing that POLH-1 and BRC-1 protect cells against radiation at very different developmental stages (see below).

**Figure 3:**
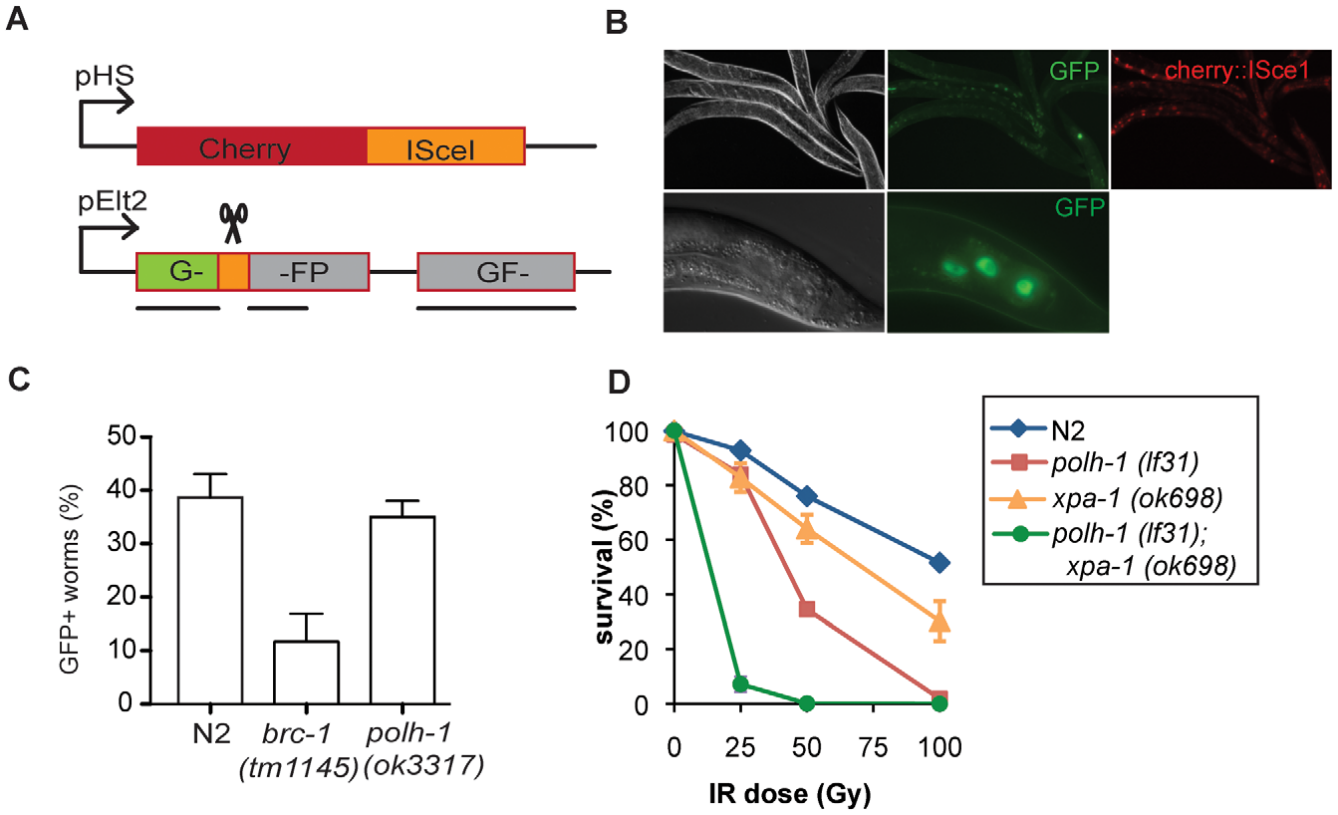
Reporter system for homologous recombination in *C. elegans*. (A) Schematic representation of the reporter transgenes. Expression of the yeast endonuclease ISceI fused to Cherry is controlled by the heat shock promotor (pHS). The reporter transgene is placed behind the intestinal *elt-2* promotor (pElt-2). Upon activation of ISceI following heat shock, the ISceI endonuclease cuts into the GFP coding sequence. Repair by gene conversion from an aborted copy of GFP results again in full length GFP. (B) Expression of GFP and Cherry in worms containing the reporter transgenes. Upon heat shock induction all intestinal cells express cherry::ISceI. Repair of the break site by HR from an aborted GFP template results in GFP expression in some intestinal cells. (C) Quantification of the fraction of GFP positive worms in different genetic backgrounds. Each bar represents the mean of three independent experiments. Error bars denote the s.e.m. (D) Germline sensitivity of *polh-1 (lf31)*, *xpa-1(ok698)* and double mutants to γ-irradiation. Each line represents the mean of minimal three independent experiments. The percentage of surviving progeny is relative to the fraction of surviving progeny without any irradiation, since *polh-1(lf31)* and *xpa-1(ok698)* show about 30% synthetic lethality. Error bars denote s.e.m.

To further test whether the sensitivity of the *polh-1* mutants to γ-irradiation is due to a possible defect in HR of DSBs, we determined the role of Polη in response to DSBs endogenously produced by DNA transposition. Transposition is desilenced in the germline of *rde-3* mutants [32] and the ensuing DSBs predominantly rely on HR for their repair [33]. However, embryonic lethality was not increased in *polh-1; rde-3* double mutants, in contrast to increased lethality in *brc-1; rde-3* doubles (Table S2). As an independent and a direct method to address a possible *in vivo* role of *C. elegans* Polη in HR, we measured repair of a site-specific DSB using a somatic HR reporter assay (Figure 3). In this assay, which will be described in more detail elsewhere, heat shock-induced expression of the yeast endonuclease I-SceI leads to a DSB in the coding sequence of a GFP transgene that is driven by the intestinal *elt-2* promoter. This transgenic setup monitors intrastrand HR, specifically in E-lineage cells, which are still proficient to enter S-phase post embryonically (in contrast to many other post embryonic cells that arrest in G1 and rely on non-homologous end-joining to repair DSBs). A functional GFP transgene is generated following DSB induction only when repair uses a downstream GFP fragment as donor sequence (Figure 3A). This outcome will manifest as GFP expressing intestinal cells. While *brc-1(tm1145)* mutation resulted in a profound reduction in the number of cells that expressed GFP, *polh-1(ok3317)* mutant animals displayed similar numbers of cells expressing GFP with similar intensities as compared to wild type worms (Figure 3B and 3C). These data further support the notion that the observed sensitivity of *polh-1* mutants to γ-irradiation is not caused by a defect in HR.

We thus explored an alternative explanation, in which the increased cytotoxicity of *polh-1* mutant animals towards γ-irradiation is the result of failed bypass of other (non-DSB) DNA lesions. Apart from DSBs, γ-irradiation induces single strand breaks (SSBs) as well as 8-Oxo-dG sites and thymine glycols [34]. We reasoned that base adducts in the DNA caused by γ-irradiation may resemble helix-distorting lesions that are substrates for NER and TLS. To address this hypothesis, we tested *xpa-1* animals as well as animals defective for both *xpa-1* and *polh-1* for sensitivity to γ-irradiation (Figure 3D). Strikingly, a redundant effect of both factors was observed after exposure to γ-irradiation similar to the effect seen after UV-irradiation (Figure S2B). These results suggest that γ-irradiation of the germline causes replication-blocking lesions that are substrates for NER and can be bypassed by Polη. It also implies that genes previously found to be involved in γ-irradiation protection are not necessarily involved in the repair of DNA breaks [35].

### Damage bypass by POLH-1 during early embryogenesis

**Figure 4:**
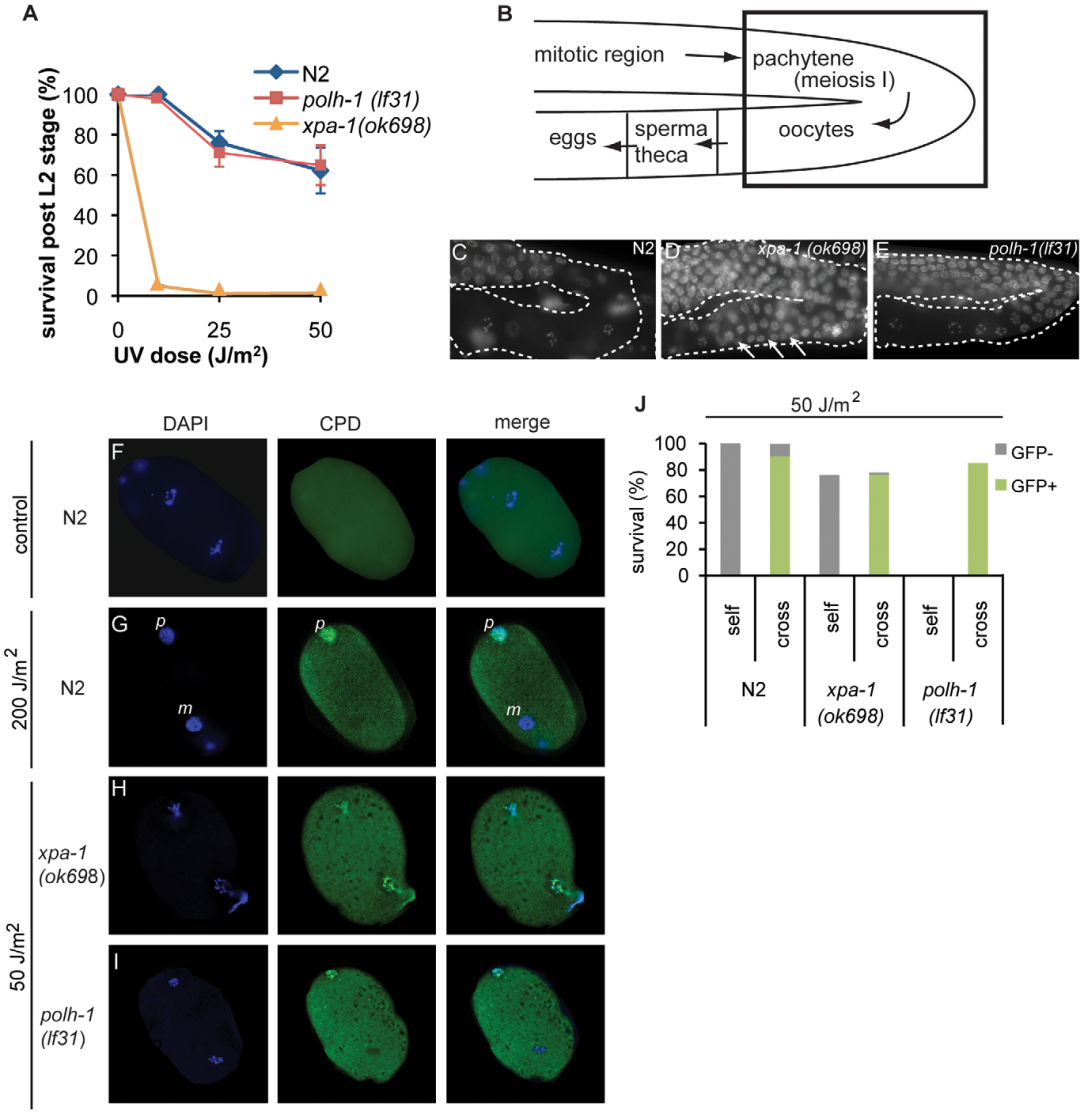
*C. elegans polh-1* and *xpa-1* adults and embryos exposed to UV at different stages during development. (A) Survival of larvae irradiated at L1 stage. Each line is the mean of three independent experiments; error bars denote s.e.m. (B) Schematic overview of the *C. elegans* germline. Boxed area shows the transition in the germline bend from the pachytene to maturating oocytes displayed in pictures C–D. (C–E) DAPI stainings of germlines of indicated genotype 16 hrs after exposure to 120 J/m^2^ UV. Morphology of the germline is completely disrupted in *xpa-1* mutants (D) but not in wildtype (C) or *polh-1* worms (E). Oocyte maturation in irradiated *polh-1* mutants is normal (E), while most meiotic cells fail to progress into oocytes in *xpa-1* worms after UV-irradiation (D), causing expansion of the pachytene region through the germline bend (arrows). (F–I) Presence of CPDs during the first embryonic divisions. Immunofluorescence on just fertilized N2 embryos 24 hrs after treatment with 200 J/m^2^ shows that only one of the two pronuclei carries CPDs (F and G). In UV irradiated *xpa-1* embryos both pronuclei carry CPDs (H) while in *polh-1* embryos, similar to wildtype (G), only one pronucleus contains CPDs (I). N.B. a lower UV dose was used in H and I to compare doses that induced similar levels of lethality. (J) UV-irradiated hermaphrodites were crossed with untreated males carrying a Pmyo-2::GFP transgene. UV-induced lethality is partly rescued in the cross progeny of *polh-1*, but not *xpa-1* hermaphrodites.

*C. elegans polh-1* mutants are far more sensitive to various DNA damaging agents as compared to vertebrate cells. We hypothesized that the dependence on POLH-1 for damage tolerance might be specific for early embryonic development, when TLS by POLH-1 is the predominant mechanism to avoid checkpoint activation by replication fork blocks on damaged DNA [6]. In differentiated cells, NER or other repair pathways may dominate the damage response. We therefore tested at which stage during development of *C. elegans* either POLH-1 mediated damage bypass or NER dominate the response to UV-irradiation. First, we exposed synchronized larvae of the L1 stage to UV light and quantified survival and growth (Figure 4A and Figure S3A). L1 larvae already contain 558 of the total 959 somatic cells that make up the adult animal, and thus mainly grow by cellular volume expansion as opposed to mitotic proliferation [36]. Although *xpa-1* mutants completely arrest in L1 after a low dose of UV (Figure 4A, [27]), in *polh-1* mutants L1 development is only slightly delayed (Figure S3A). Ultimately *polh-1* mutants displayed similar survival as found for wildtype L1s following UV exposure (Figure 4A), indicating that in contrast to XPA, POLH-1 plays hardly any role in the UV damage response in L1. Second, we found that germ cell maturation in *polh-1* mutants was comparable to wildtype following UV exposure (Figure 4B–4E), in contrast to *xpa-1* mutants that (i) display an UV-induced expansion of the pachytene region and (ii) fail to generate normal-sized oocytes (Figure 4D), [27]. In addition we determined the apoptotic response in the germline after UV irradiation using a *ced-1::GFP* transgene that marks germ cells in the process of apoptosis [37]. In contrast to *xpa-1* deficient animals [38], we found no reduction in the UV-dependent apoptotic response in *polh-1* mutants as compared to wildtype animals (Figure S4). Together, these data indicate that NER is essential for normal gametogenesis and L1 development following UV exposure. Apparently, in *polh-1* mutants there is sufficient time for repair of UV lesions in these developmental stages to prevent replication stress.

However, limited time for DNA repair is available immediately upon fertilization, when a *C. elegans* embryo goes through a 3-hour period of rapid divisions, according to a fixed and time-constrained lineage program [31]. Thus, in this developmental stage incomplete removal of DNA damage could account for the severe embryonic lethality of UV-exposed *polh-1* mutants. To test this hypothesis, we studied the persistence of CPDs - the most abundant lesion type caused by UV – in pronuclei of oocytes, just after fertilization. We irradiated adults with 200 J/m^2^ and after 24 hours stained developing embryos for CPDs. Remarkably, in wildtype embryos CPDs were still present in the paternal pronucleus, while no CPDs were observed in the maternal pronucleus (Figure 4F–4G). We next assayed *xpa-1* and *polh-1* mutants after a dose of 50 J/m^2^ (leading to comparable levels of embryonic lethality). Mutants defective in *xpa-1* displayed CPD staining in both pronuclei (Figure 4H), suggesting that in wildtype animals NER-dependent removal of CPDs has occurred during meiotic maturation of the germ cells. In contrast to *xpa-1* mutants, but similar to wildtype animals, *polh-1* mutants were proficient in removal of CPDs from the maternal pronucleus, whereas CPDs were clearly detectable in the paternal pronucleus (Figure 4I). Before migration and fusion with the maternal pronucleus, the paternal genome decondenses and is replicated in less than 12 minutes [39]. This time span is insufficient for NER to remove DNA damage. We hypothesize that the presence of unrepaired damage from the paternal DNA poses a problem on the first mitotic divisions in *polh-1* early embryos. To address this hypothesis, we mated UV-irradiated wildtype or mutant hermaphrodites with untreated males, providing a source of undamaged sperm DNA (Figure 4J). To mark the progeny we used a transgenic line expressing Pmyo-2::GFP. Indeed, lethality in the progeny of irradiated *polh-1* hermaphrodites is almost fully rescued by providing a source of undamaged sperm DNA. In contrast, mating of *xpa-1* hermaphrodites with untreated males does not affect survival of the progeny. Together, these data indicate that correct progression of early embryonic cell divisions strongly relies on POLH-1 when the genome contains DNA damage. This dependency is not restricted to UV-induced damage but also extends to DNA damage induced by γ-irradiation. The increased sensitivity of *polh-1* mutants to γ-irradiation can also be completely rescued by crossing irradiated hermaphrodites with untreated males, thus providing a non-damaged paternal genome (Figure S5). Importantly, this is in stark contrast to the sensitivity of *brc-1* mutants, which cannot be rescued by providing non-damaged sperm. This developmental separation of the modes of action of these proteins further substantiates our findings that *polh-1* and *brc-1* act independently in protecting cells against γ-irradiation-induced DNA damage.

### POLH-1 and POLK-1 act in a redundant fashion in protection against the methylating agent MMS

**Figure 5:**
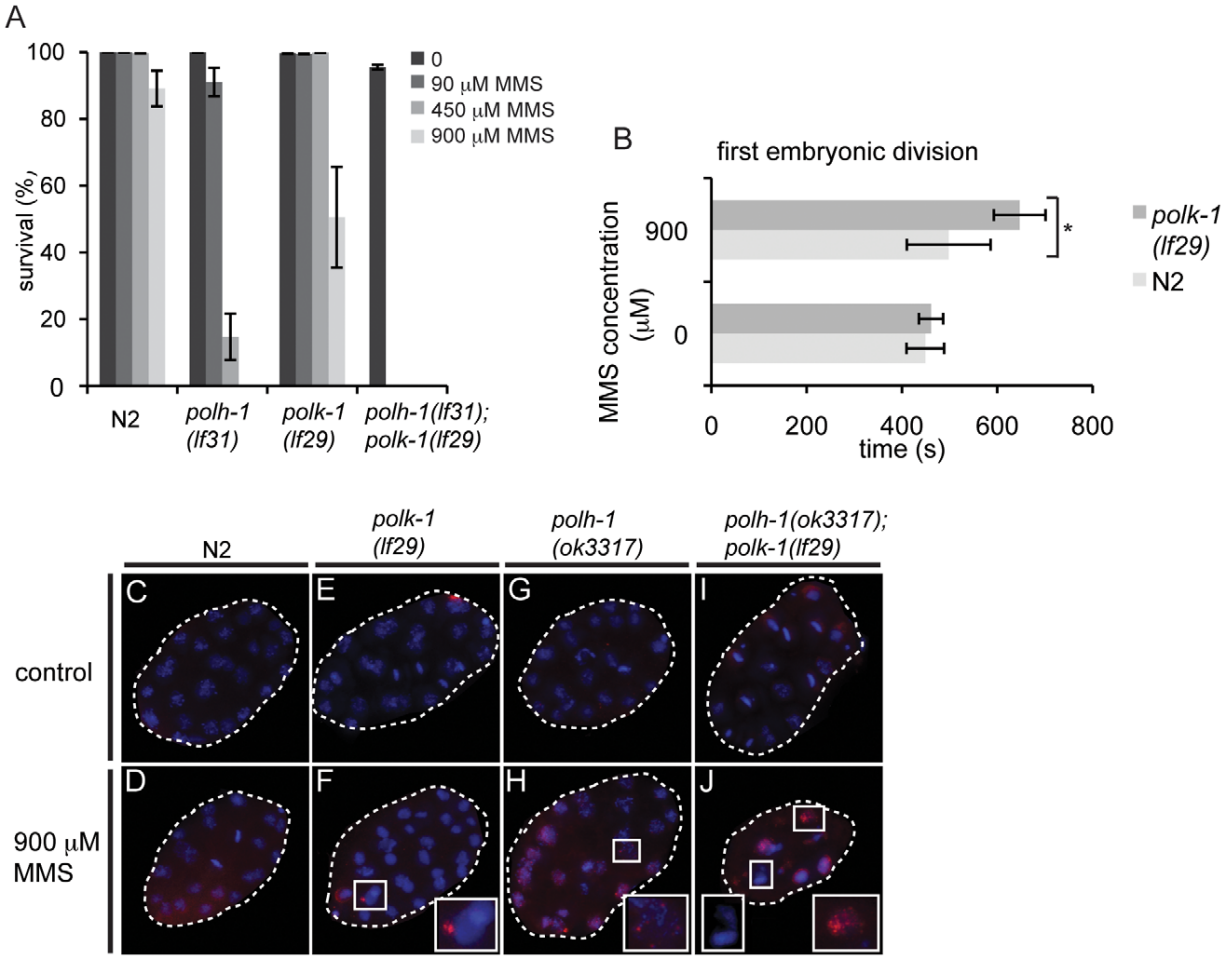
*polh-1* and *polk-1* protect in a redundant fashion against the methylating agent MMS. (A) Double mutants of *polh-1* and *polk-1* are severely sensitized to MMS exposure. Results of a representative experiment are shown. Error bars denote SD. (B) Delayed progression through the first embryonic division after MMS exposure in *polk-1* mutants. The interval between passing of the paternal pronucleus over the midline till the start of cytokinesis is timed for at least 5 embryos per datapoint. Statistical significance for the difference in delay between N2 and *polk-1* embryos treated with MMS was calculated with a student's t-test (p = 0.012). (C–J) RAD51 immunostainings of early embryos treated with MMS. Morphology of *polh-1; polk-1* double mutant embryos is abnormal after MMS exposure, displaying chromatin bridges and abundant RAD51 staining (J). *polh-1(ok3317)* and *polk-1(lf29)* single mutants show incidental RAD51 foci in embryos (F and H), while such foci were never observed in untreated controls (C, E, G and I).

We next wondered whether a similar developmentally restrained function could be attributed to TLS polymerase POLK-1. To address this question, we exposed *polk-1(lf29)* mutant worms to different doses of UV, cisplatin or γ-irradiation (Figure 2A–2C), but found no difference in sensitivity as compared to wildtype animals, indicating that POLK-1 is not involved in protection against these sources of DNA damage in *C. elegans*. However, akin to the outcome of published RNAi experiments [6], both *polk-1* and *polh-1* mutants are sensitive to chronic exposure to the alkylating agent MMS, albeit that the sensitivity in *polh-1* mutants was much more pronounced (Figure 5A, Figure S6), indicating that both POLH-1 and POLK-1 play a role in bypass of MMS-induced DNA damage. We next assayed *polh-1 (lf31); polk-1 (lf29)* double mutants and *polh-1 (ok3317) polk-1*(RNAi) animals for MMS sensitivity (Figure 5A, Figure S6A). Interestingly, double mutants were extremely sensitive to MMS, and complete lethality was observed at a dose that was 100 times lower than the effective dose for any of the single mutants (Figure 5A, Figure S6A). We did not observe any synergistic effect for any of the other types of lesions we tested (Figure 2).

POLH-1 has previously been shown to be involved in avoiding DNA damage-induced checkpoint activation [6]. In *C. elegans* embryogenesis, checkpoints - mediated by the *C. elegans* homologs of the checkpoint genes ATR and CHK-1 - are used to time the first asynchronous cell divisions that are essential for embryonic patterning and thus embryonic viability [5]. Checkpoint activation due to DNA damage interferes with the developmental role of the checkpoint, causing patterning defects and embryonic lethality. Our results with null mutants for *polh-1* and *polk-1* suggest that both POLH-1 and POLK-1 can act to avoid checkpoint activation. To test the involvement of POLK-1 in checkpoint avoidance directly, we timed the first embryonic division of *polk-1* embryos after exposure to MMS. Figure 5B illustrates a delayed first embryonic division in *polk-1* mutants when compared to wildtype embryos. Importantly, we also observed examples of *polk-1* embryos that after MMS treatment fully arrested at the 1-cell stage (Videos S1 and S2), while we never observed such cases for MMS-treated wildtype embryos. Two other phenotypes are also indicative of replication stress during early embryonic divisions of MMS treated *polh-1* and *polk-1* mutant animals. First, *polh-1; polk-1* double mutant embryos displayed foci of the DSB repair marker RAD51 (Figure 5C–5J), indicative of DSBs resulting from trying to replicate damaged genomes [40]. Second, DAPI staining revealed chromatin bridges and a disrupted nuclear morphology in the early embryo (Figure 5J), suggesting division of disentangled or incompletely replicated genomes. These phenotypes were less profound, but noticeable, in both single mutants, while never observed in wild type embryos exposed to similar MMS concentrations (Figure 5C–5H).

To investigate whether the dependency on POLH-1 and POLK-1 for tolerance to MMS was restricted to embryogenesis - similar to the requirement of POLH-1 in UV tolerance - we followed the outgrowth of L1 animals exposed to increasing concentrations of MMS (Figure S6B). The development of *polh-1* larvae was mildly affected, while no delay was observed for *polk-1* animals. As for UV, NER deficient *xpa-1* larvae were profoundly more sensitive to MMS than either *polk-1* or *polh-1* deficient larvae (Figure S6B), while the opposite is true for embryonic stages: *xpa-1* embryos are less sensitive to MMS than *polh-1* embryos [6]. This again argues that TLS is more important than DNA repair at developmental stages that are characterized by fast replication cycles.

### A genetic approach for identifying new factors in TLS regulation in the early embryo

**Figure 6:**
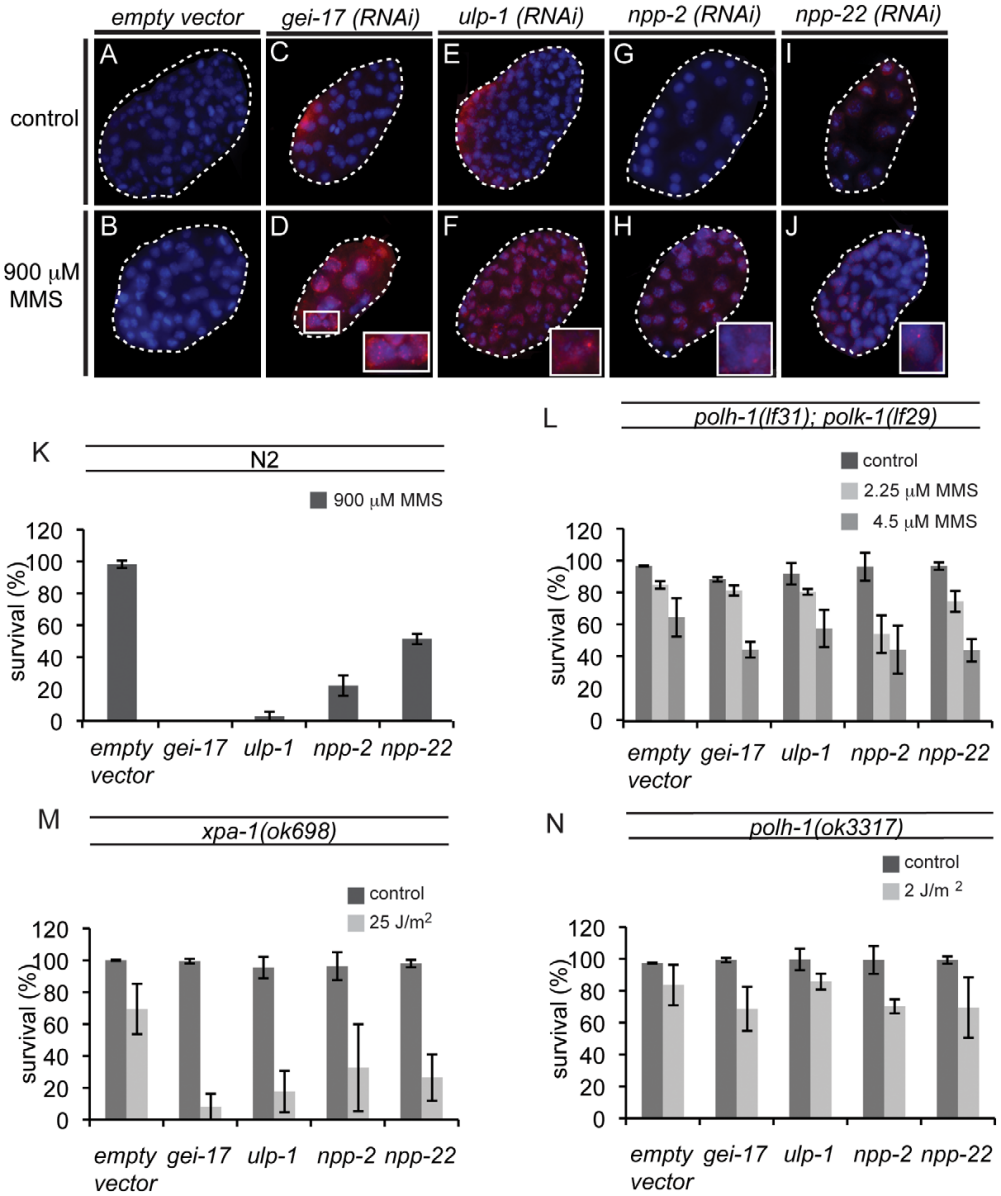
New factors in damage tolerance in the early embryo. (A–J) RAD51 stainings of early embryos treated with MMS. *gei-17* RNAi knockdown embryos show abundant RAD51 staining after treatment with MMS (D). Incidental RAD51 foci are observed in *ulp-1*, *npp-2 and npp-22* knockdown embryos (F, H, J) but not in wildtype controls (B). (K) MMS sensitivity of *gei-17*, *ulp-1*, *npp-2* and *npp-22* knockdowns. (L) Sensitivity to MMS is not further reduced in *polh-1; polk-1* mutants by any of the tested RNAi clones. (M) Sensitivity to UV is further reduced by indicated RNAi food against *gei-17*, *ulp-1*, *npp-2* and *npp-22* in a *xpa-1* mutant background. (N) Sensitivity to a low dose of UV is not further reduced in a *polh-1* mutant background after knockdown by indicated RNAi foods.

Since POLH-1 and POLK-1 together appear to be extremely important in protecting the developing embryo against MMS, we wondered whether there might be a general pathway underlying the regulation of the two TLS enzymes. To identify new factors regulating TLS in the early embryo, we performed a genome-wide RNAi screen for genes sensitizing embryos to MMS. Out of 16,757 genes tested (covering ∼86% of all predicted *C. elegans* genes), we found 87 genes that resulted in sensitivity to MMS upon knockdown, including *polk-1*. *polh-1* was not identified in this screen, probably due to insufficient knockdown by the RNAi clone targeting this gene. We next inspected these RNAi knockdowns for phenotypes reminiscent of *polh-1;polk-1* double mutants. All 87 hits were analysed by DAPI for altered nuclear morphology after exposure to MMS (Figure 5A and Figure S7). Four clones were selected for follow-up analysis based on perturbed embryonic divisions as indicated by chromatin bridges and malformed nuclei. These clones targeted the genes *gei-17, ulp-1, npp-2* and *npp-22* (Figure 6). *gei-17* encodes a SUMO-protease that was previously shown to interact with POLH-1 after DNA damage [13]. *ulp-1* encodes a ubiquitin-like protease (ULP) that deconjugates SUMO moieties from their target proteins [41]. *npp-2* and *npp-22* encode two components of the *C. elegans* nuclear pore complex (NPC)[42]. Null alleles of *gei-17*, *npp-2* and *npp-22* are embryonic lethal. Knockdowns of the four genes reduced tolerance to MMS to a similar extent as mutations in *polh-1* and *polk-1* (Figure 6K). In line with published data [6] we found that *gei-17* knockdown led to abundant RAD51 foci in embryos treated on MMS, indicative of replication stress. Also knockdown of *ulp-1*, *npp-2* and *npp-22* lead to MMS-induced RAD51 foci, although to a lesser extend than *gei-17* knockdown. This is consistent with the observation that these knockdowns also display less dramatic effects on progeny survival. Foci formation was never observed in mock-treated knockdowns or wild type controls (Figure 6A–6J).

We hypothesized that if these genes were in a common pathway with POLH-1 and POLK-1, then knockdown of these factors would not further increase sensitivity of *polh-1;polk-1* worms to a low dose of MMS. Indeed, MMS sensitivity was not further increased when *ulp-1*, *gei-17, npp-2* and *npp-22* were knocked down in *polh-1;polk-1* double mutant animals (Figure 6L), placing all four factors in an epistatic relation to the TLS genes in the response to alkylating damage. To substantiate this epistatic relationship we also studied another source of DNA damage infliction, by exposing young adults to UV light. We previously showed that *polh-1* is important for embryonic development in the presence of UV damage, and that an additional mutation in the NER factor *xpa-1* renders the animals even more sensitive to low doses of UV (Figure 2A and Figure S2B). We argued that if these factors act in a common pathway with Polη in the response to UV, we would expect their knockdowns to be epistatic with a *polh-1* mutation, but increase the sensitivity of *xpa-1* defective animals. Indeed, knockdowns of *gei-17, ulp-1, npp-2* or *npp-22* all further increased the sensitivity of *xpa-1* mutant animals to a low dose of UV (Figure 6M), but did not change sensitivity of *polh-1* mutants (Figure 6N). Together these data indicate that *ulp-1, npp-2* and *npp-22* are novel factors in TLS mediated by POLH-1 and POLK-1 in response to DNA damage during early embryogenesis in *C. elegans*. The SUMO protease gene *gei-17* has previously been shown to promote damage tolerance by sumoylating POLH-1 [13]. Our results suggest that GEI-17 is implicated in TLS mediated by both POLH-1 and POLK-1.

## Discussion

Here we demonstrate that there is modulation of the choice between repair and bypass of damaged template DNA in a developing organism. *A priori* one would expect error-free repair by NER to be the favoured option in germ cells to prevent the accumulation of mutations in subsequent generations. Indeed, we and others found that both for germ cell maturation and post-embryonic somatic development, NER is indispensable in response to specific DNA damages [26], [27], [38]. However, and in line with previously published data [6], we found that immediately after fertilization of the oocyte, during stages of rapid cell divisions in the early embryo, survival is determined by TLS factors and not by NER. The need for efficient TLS must be viewed in light of the strict timing of the developmental program, which likely does not allow time to “wait” for repair processes to be completed. Our observation that wild type animals can easily survive UV doses up to 50 J/m^2^ without substantially repairing CPDs from their sperm or decondensed paternal pronucleus indicates that TLS-proficient zygotes can replicate a damaged genome containing 10–50.000 UV lesions in less than 12 minutes - the time it takes for the paternal nucleus to double its genome content - without delaying cell division [39].

We found that *C. elegans* POLH-1 has a broader substrate specificity than POLK-1; POLH-1 is involved in bypass of damage induced upon exposure to UV light, γ-irradiation, cisplatin and MMS. We considered the possibility that all treatments may lead to a common substrate that causes the observed cytotoxicity, such as DSBs brought about by replication fork obstruction and collapse. This notion has been supported by studies in vertebrates, in which Polη was suggested to act in HR repair of DSBs by extending the D-loop intermediate structure [11], [12]. However, we observed a wild type response to either transposon-mediated or ISceI-induced DSBs, thus arguing against a role for POLH-1 in DSB repair, in either germline or somatic tissue of *C. elegans*. We ascribe the sensitivity of *polh-1* mutants towards γ-irradiation to the induction of other non-DSB lesions, which may be NER substrates. Consistent with this interpretation we observed a synergistic relationship between *xpa-1* and *polh-1* with respect to IR sensitivity. The induction of free radicals by ionizing radiation causes a plethora of lesions in the DNA, such as 8-oxo-dG sites, which may require Polη-mediated bypass to prevent checkpoint activation [43].

An explanation for the broad substrate specificity of POLH-1 may reside in the flexible active site of POLH-1, which may allow for bypass of lesions that are structurally very different. Indeed, studies in chicken DT40 cells indicate that Polη is a much more versatile polymerase than the phenotype of XPV cells would suggest [44]. Alternatively, Polη could have an indirect role by recruiting other TLS proteins to the damage site. In human cells Rev1 is recruited to UV damages *via* an interaction with Polη [45]. Interestingly, Polκ has been shown to serve as a ‘backup’ polymerase in XPV cells in the bypass of both UV-induced CPDs as well as cisplatin adducts [46], [47]. We here show that in nematodes this genetic interaction is restricted to damage induced by the S_n_1 methylating agent MMS. The molecular effects of MMS include the formation of N-7 methylguanine (which by spontaneous depurination can lead to an abasic site), N3- methyladenine, N3-cytosine and O^6^-methylguanine [48]. Although we cannot deduce from our in vivo analysis which of these damages underlies the cytotoxicity observed in nematodes, all of these base damages have residual coding capacity, and are less structurally perturbing than some of the DNA lesions induced by cisplatin or IR treatment. This notion may explain the redundant role of the functionally more restricted POLK-1 on MMS, while no contribution was seen following UV, IR or cisplatin treatment.

In order to find novel factors that are directly or indirectly involved in TLS, we screened for RNAi knockdowns that rendered cells sensitive to MMS and UV, only in the context of TLS functionality. Out of ∼17,000 clones we identified four genes whose knockdown sensitized wildtype but not TLS-deficient animals to MMS treatment. One of these genes, *gei-17*, was previously reported to regulate Polη; GEI-17 was shown to sumoylate POLH-1 near its PIP-box motif resulting in protection of the protein from degradation [13]. The profound effects of *gei-17* RNAi on cellular tolerance to MMS suggest that this SUMO-ligase most likely acts on both POLH-1 and POLK-1 (Figure 5A and 5I); we note that *C. elegans* POLK-1 may also contain a PIP-box motif (Figure S1). In addition to GEI-17 we also identified the SUMO protease ULP-1 as a factor in TLS-mediated MMS- and UV-sensitivity. This result suggests that, apart from sumoylation, also desumoylation may play a role in the regulation of TLS proteins. Additional studies that identify targets of ULP-1 needs to establish whether its role is direct, by desumoylation of the TLS polymerases, or indirect. Ubiquitin-like proteases (ULPs) deconjugate SUMO from their target proteins and therefore the damage sensitivity of ULP-1 knockdown may also be explained by disturbed regulation because of a shortage of SUMO. SUMO proteases and ligases may anchor to the NPCs in order to sumoylate or desumoylate their targets [49]. Here we show that, similar to *gei-17* and *ulp-1*, RNAi against nuclear pore components *npp-2* and *npp-22* is compatible with viability but results in sensitivity to UV lesions and MMS. However, sensitivity was not further increased in the absence of POLH-1 and POLK-1. This finding suggests a role for the NPC (or NPC subunits) in TLS mediated damage tolerance, possibly in the localization of SUMO-regulation. *npp-2* encodes the *C. elegans* homolog of yeast Nup85, which is one of the proteins of the scNup84 scaffolding complex. In yeast, mutants in the Nup84 and Nup60/Mlp1-2 complexes have similar phenotypes in the response to DNA damage as Ulp1 mutants [50]. A direct link of NPCs to the DNA damage response in yeast was also suggested by Nagai et al., who showed relocation of damaged DNA to nuclear pores [51] and recently by Bermejo et al. who showed involvement of inner basket proteins in replication fork stability [52]. *npp-22* encodes the *C. elegans* homolog of yeast and mammalian NDC1, which is crucial for nuclear pore assembly [53]. Future work on *gei-17*, *ulp-1* and the nuclear pore components *npp-2* and *npp-22* is needed to substantiate the role of sumoylation and desumoylation processes and a possible link to the NPC (subunits) in regulating TLS.

## Materials and Methods

### C. elegans genetics

All strains were cultured according to standard methods [54]. Wildtype N2 (Bristol) worms were used in all control experiments. The *polh-1 (lf31)* and *polk-1 (lf29)* mutants were isolated in our own laboratory. *polh-1 (ok3317)* worms, that were kindly provided by Joel Meyer (Duke University, Durham NC, USA), have been generated by the *C. elegans* knock-out consortium. *BCN2081*, carrying a single copy integrated Pmyo::GFP transgene, was a gift from Ben Lehner (EMBL Centre for Genomic Regulation, Barcelona, Spain) [55]. All other alleles *(xpa-1 (ok698)*; *rde-3 (ne298); brc-1 (tm1145)*; *dog-1 (gk10))* and the transgenic line MD701 *(bcIs39[P(lim-7)ced-1::GFP+lin-15(+)])* were obtained from the *C. elegans* Genetics Center (St Paul, MN, USA). All mutant strains were backcrossed six times before performing experiments. Newly generated strains are listed in Table S1 in the supplementary information.

### Survival assays

Staged animals were exposed to different doses of various DNA damaging agents. To assess germline sensitivity three plates with three worms were allowed to lay eggs for 24–48 hrs per experimental condition. 24 hrs later, the number of unhatched eggs and the number of surviving progeny was determined. All experiments were performed in triplicate. To measure germline sensitivity to UV, staged young adults one day post L4 were transferred to empty NGM plates and exposed to different doses of UV-C (predominantly 254 nm, Philips). Animals were placed on fresh OP50 plates and allowed to lay eggs for 32 hrs.

To determine whether lethality could be rescued by the supply of undamaged sperm, UV irradiated hermaphrodites were mated with untreated BCN2081 worms, which have Pmyo-2::GFP transgenes integrated in their genomes. After 24 hrs of male contact, the hermaphrodites were transferred to individual plates and allowed to lay eggs for 24 hrs. The mother was subsequently removed and 24 hrs later the number of non-hatched eggs and the number of GFP+ and GFP- progeny was determined.

The sensitivity of L1 larval stage animals to UV was measured as described previously [27]. L1s were synchronized by bleaching, and exposed to UV-C on empty NGM plates. Per plate, at least 100 L1 animals were counted. For three subsequent days the development of L1-treated animals was monitored.

To measure germline sensitivity to γ-irradiation, different doses were delivered by an X-ray generator (dose rate 7 Gy/min; YXLON International) to L4 animals. Animals were allowed to lay eggs for 48 hrs, and scored 24 hrs later for hatching.

Sensitivity to cisplatin was determined by incubating staged L4 animals for 3 hrs in M9 containing different concentrations of cisplatin (Sigma-Aldrich). After 1 hr recovery on OP50 plates, animals were placed on fresh OP50 plates and allowed to lay eggs for 48 hrs. The mother was removed and the survival of the progeny was scored 24 hrs later.

To measure sensitivity to chronic exposure to MMS, staged L4 animals were placed for 24 hrs on NGM plates containing different concentrations of MMS (Sigma-Aldrich). After 24 hrs, the number of non-hatched eggs and surviving progeny was determined.

### Homologous recombination (HR) assay

A HR reporter plasmid was constructed consisting of a GFP/LacZ fusion under the control of the intestinal specific *elt-2* promotor [56]. An ISceI recognition sequence was inserted that disrupted the GFP ORF. To provide a template for homologous recombination, part of the GFP coding region was PCR amplified and inserted downstream of the disrupted GFP/LacZ locus. The ISceI expressing plasmid pRP3001 (hsp-16.41::ISceI ORF) [57], was modified to include the mCherry ORF leading to a functional ISceI/mCherry protein to visualize and monitor the expression of the ISceI endonuclease. A detailed description of the reporter system and its validation will be published elsewhere.

For reading out HR, synchronized L4 animals were transferred and incubated for 1.5 hrs at 34°C. After 24 hrs, GFP expression in the intestine was analyzed on a Leica DM6000 microscope.

### Microscopy

Nuclear stainings on germlines and embryos were performed by incubation of staged young adults for 10 minutes in ethanol containing 10 µg/mL 4′,6-diamidino-2-phenylindole (DAPI). After two washes with PBS, worms were mounted on object slides in 30% glycerol.

To detect CPDs, eggs were liberated from UV-irradiated worms and fixed with 3% paraformaldehyde. Fixed eggs were permeabilized by freeze cracking and subsequently washed with 1% Triton and methanol (−20°C). CPDs were visualized by subsequent staining with an anti-CPD mouse monoclonal antibody and an Alexa488-labelled goat-anti-mouse secondary antibody (Molecular Probes Inc) combined with 10 µg/mL DAPI. Dissected worms and eggs were mounted on object slides in Vectashield.

To study RAD51 foci formation, a similar procedure as described for CPD staining was followed. Fixed eggs were permeabilized by freeze cracking and subsequently washed with 1% Triton and methanol (−20°C). RAD51 was visualized by subsequent staining with an anti-RAD51 rabbit monoclonal antibody and an Alexa488-labelled goat-anti-rabbit secondary antibody (Molecular Probes Inc) combined with 10 µg/mL DAPI. Dissected worms and eggs were mounted on object slides in Vectashield.

For the analysis of apoptosis, transgenic MD701 animals, expressing a *ced1*::GFP fusion behind a *lim-7* promotor, were used to visualize sheath cells surrounding apoptotic germ cells [37]. All microscopy was performed with a Leica DM6000 microscope.

### RNAi screen and RNAi experiments

Using the Ahringer Lab RNAi feeding library a genome-wide screen was performed for clones sensitizing animals to MMS. The procedure is an adaptation from a genome-wide RNAi screen for radiation sensitivity by Van Haaften et al, described in detail in their supplementary data [35]. Briefly, L1 worms were grown to L4s in liquid on RNAi food. At the L4 stage MMS was added to a concentration of 0.01%. After three days survival of the progeny was scored by visual inspection. For knockdown of *polk-1, gei-17, ulp-1, npp-2* and *npp-22* genes, individual Ahringer clones were grown on IPTG containing NGM plates. Staged L4s were transferred to RNAi plates; analysis was performed on the progeny of these animals.

## Supporting Information

Figure S1 Full alignment of *C. elegans* POLH-1 and POLK-1 with human Polη and Polk and yeast Rad30.
(TIF)

Figure S2 Germline sensitivity of *polh-1 (lf31)* and *polh-1 (ok3317)* mutants combined with repair defective backgrounds to different sources of DNA damage. (A–B) sensitivity to UV irradiation. (B) Epistasis analysis for *xpa-1* and *polh-1*. (C) Sensitivity to cisplatin. (D–F) Sensitivity to γ-irradiation. (A)(C)(D) Both alleles of *polh-1* lead to equal sensitivity to various damaging agents. (E–F) Epistasis analysis of *polh-1 (lf31)* and *brc-1(tm1145)* mutants for γ-irradiation. Data have been normalized for reduced survival (about 95%) in *polh-1;brc-1* double mutants without treatment. Results of representative experiments are shown for A, C, D and F. Error bars denote SD. Each line in B and E represents the mean of minimal three independent experiments. Error bars denote the s.e.m.
(TIF)

Figure S3 Development of L1 larvae three days after treatment with indicated UV doses. Percentage of animals in the different larval stages (L1-L2-L3-L4-young adult) was quantified 72 hrs after exposure to UV-irradiation.
(TIF)

Figure S4 Apoptosis in the germline after UV-irradiation. (A) A transgenic line expressing pLim7::ced1::GFP is used. Examples of CED1-GFP engulfed cells in the germline bend are indicated with arrows. (B) Quantification of CED1-GFP engulfed cells. In the *polh-1(ok3317)* mutant apoptosis is slightly increased after exposure to UV irradiation. About 40 germlines have been analysed per sample. Error bars denote s.e.m.
(TIF)

Figure S5 γ-irradiated hermaphrodites were crossed with non-irradiated males carrying a Pmyo-2::GFP transgene. Lethality induced by γ-irradiation is almost fully rescued in the cross progeny of *polh-1*, but not *brc-1* hermaphrodites.
(TIF)

Figure S6 POLH-1 and POLK-1 in cellular tolerance to MMS during embryogenesis and L1 outgrowth. (A) MMS sensitivity of N2 and *polh-1(ok3317)* mutants with or without depletion of POLK-1 by RNAi. (B) Development of larvae exposed to MMS from L1 stage. Each line is the mean of three independent experiments; error bars denote s.e.m.
(TIF)

Figure S7 Morphological defects in MMS exposed embryos. DAPI-stainings of whole animals exposed for 24 hrs to MMS reveal a delay in development on indicated RNAi foods. Incidentally, chromatin bridges are visible (arrows) indicative of incomplete DNA replication.
(TIF)

Table S1 List of newly generated strains used in this study.
(DOC)

Table S2 *polh-1* does not influence lethality in a mutant background where transposition is desilenced. Double mutants for the HR gene *brc-1* and the mutator gene *rde-3* display synthetic lethality while *polh-1; rde-3* double mutants are comparable to *rde-3* single mutants.
(DOC)

Video S1 First embryonic division of a *polk-1(lf29)* embryo.
(MOV)

Video S2 First embryonic division of a *polk-1(lf29)* embryo from a worm exposed for 24 hrs to MMS. Embryo arrests in the first cellular division.
(MOV)
