# Supplementary figures and images for: A Broad Requirement for TLS Polymerases η and κ, and Interacting Sumoylation and Nuclear Pore Proteins, in Lesion Bypass during C. elegans Embryogenesis
Source: PLoS Genet. 2012 Jun 28;8(6):e1002800. doi: 10.1371/journal.pgen.1002800 (PMC3386174; doi:10.1371/journal.pgen.1002800)

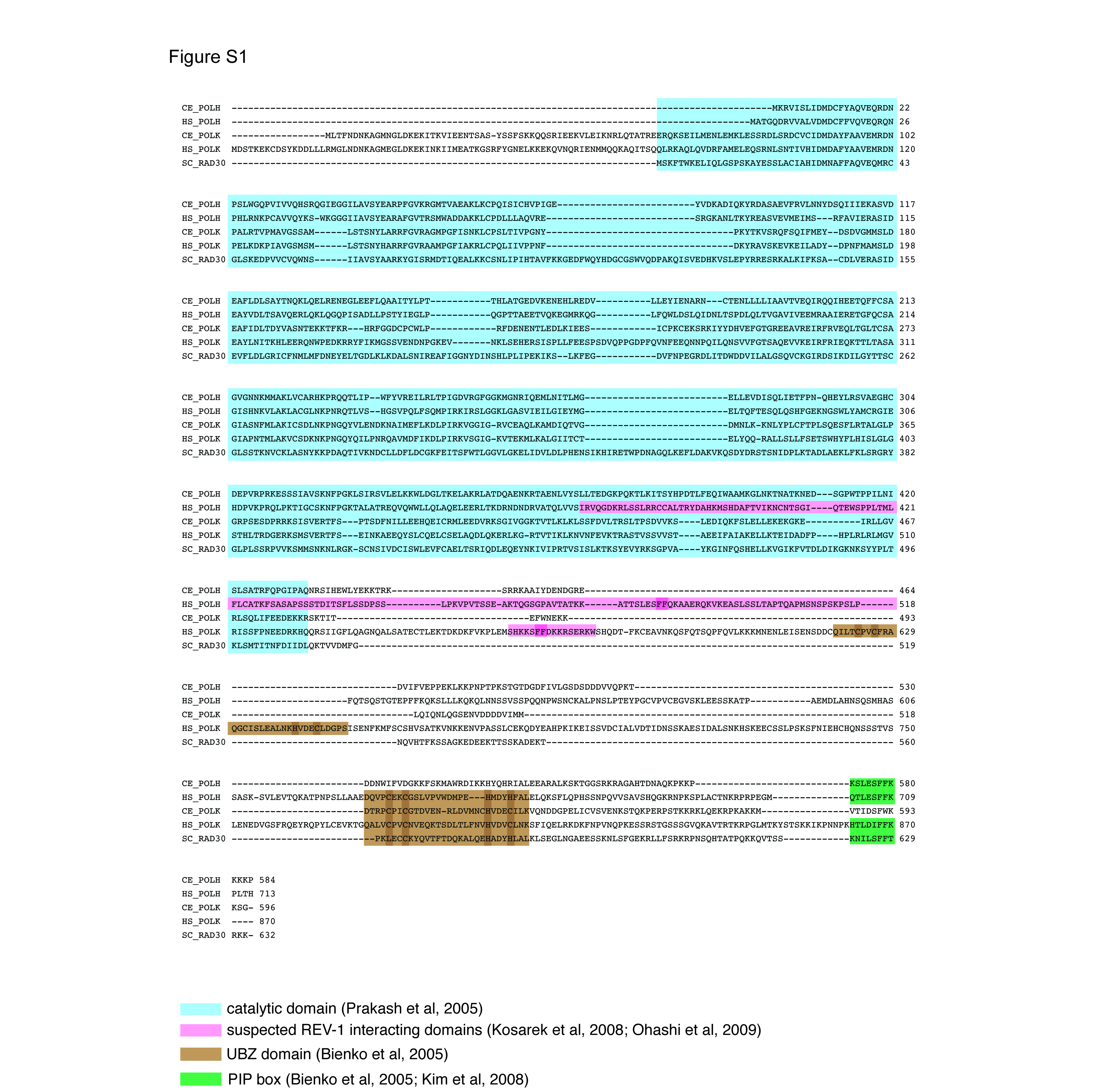

Supplement: Figure S1 — Full alignment of C. elegans POLH-1 and POLK-1 with human Polη and Polk and yeast Rad30. (TIF) [file pgen.1002800.s001.tif]

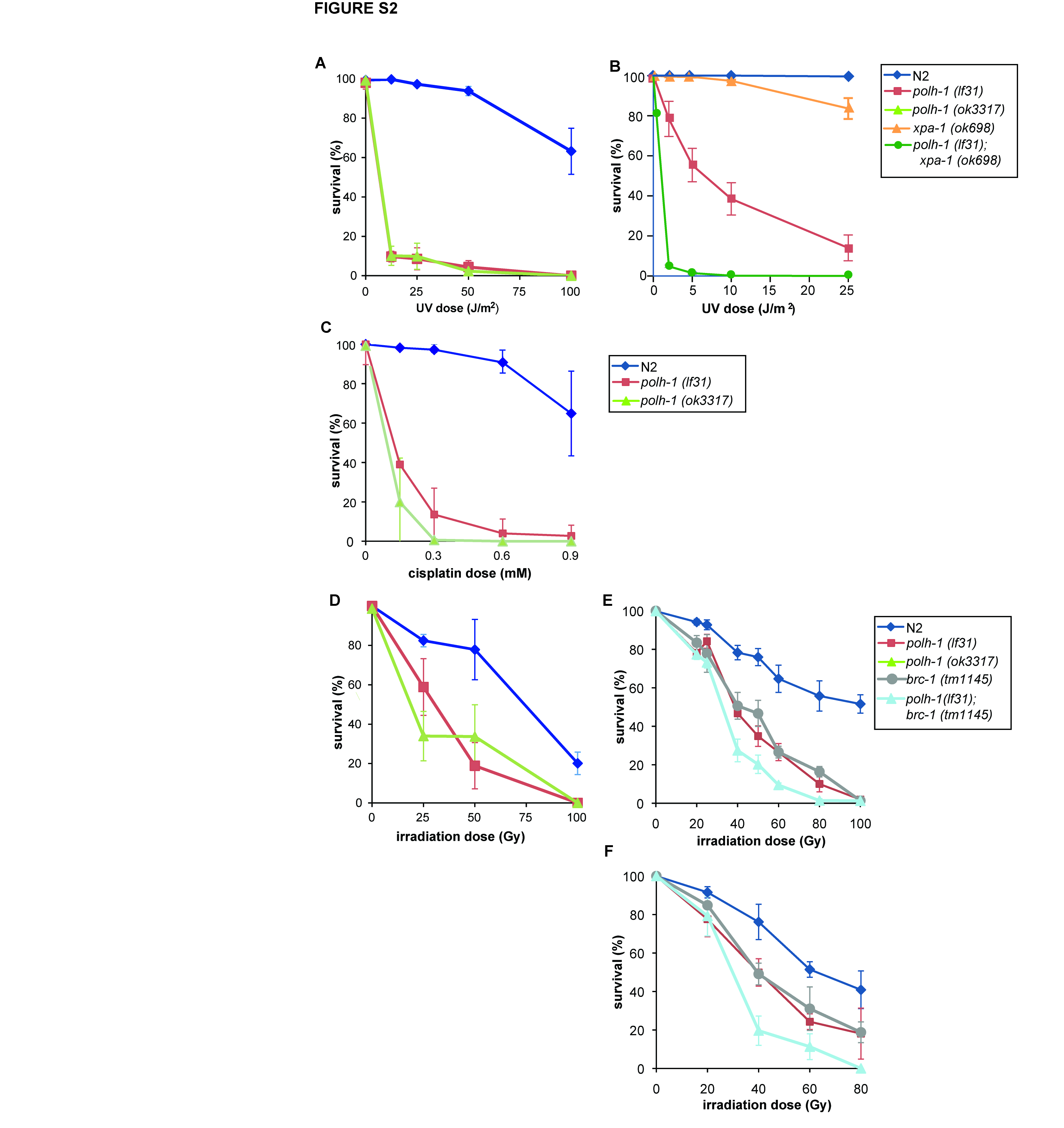

Supplement: Figure S2 — Germline sensitivity of polh-1 (lf31) and polh-1 (ok3317) mutants combined with repair defective backgrounds to different sources of DNA damage. (A–B) sensitivity to UV irradiation. (B) Epistasis analysis for xpa-1 and polh-1. (C) Sensitivity to cisplatin. (D–F) Sensitivity to γ-irradiation. (A)(C)(D) Both alleles of polh-1 lead to equal sensitivity to various damaging agents. (E–F) Epistasis analysis of polh-1 (lf31) and brc-1(tm1145) mutants for γ-irradiation. Data have been normalized for reduced survival (about 95%) in polh-1;brc-1 double mutants without treatment. Results of representative experiments are shown for A, C, D and F. Error bars denote SD. Each line in B and E represents the mean of minimal three independent experiments. Error bars denote the s.e.m. (TIF) [file pgen.1002800.s002.tif]

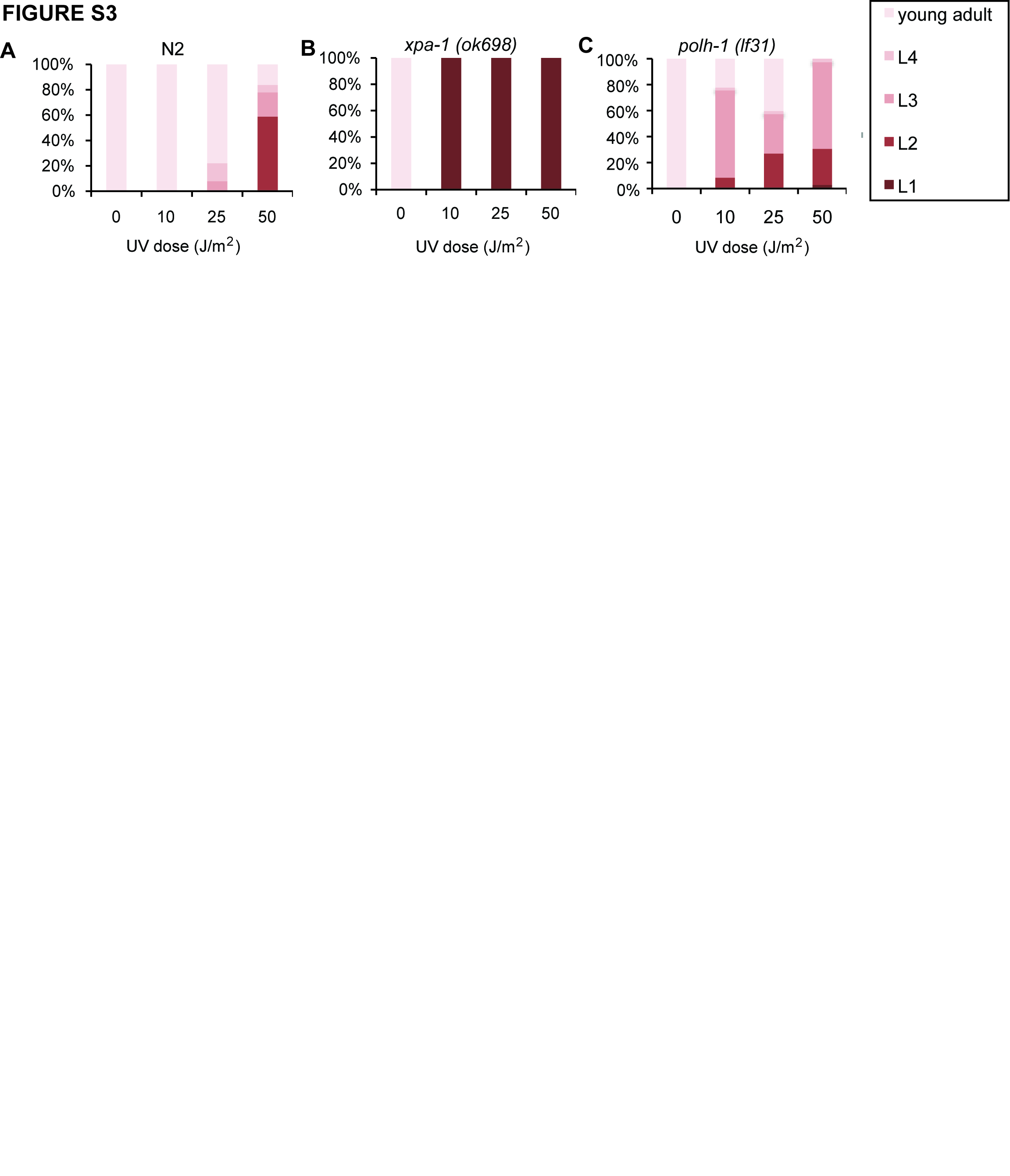

Supplement: Figure S3 — Development of L1 larvae three days after treatment with indicated UV doses. Percentage of animals in the different larval stages (L1-L2-L3-L4-young adult) was quantified 72 hrs after exposure to UV-irradiation. (TIF) [file pgen.1002800.s003.tif]

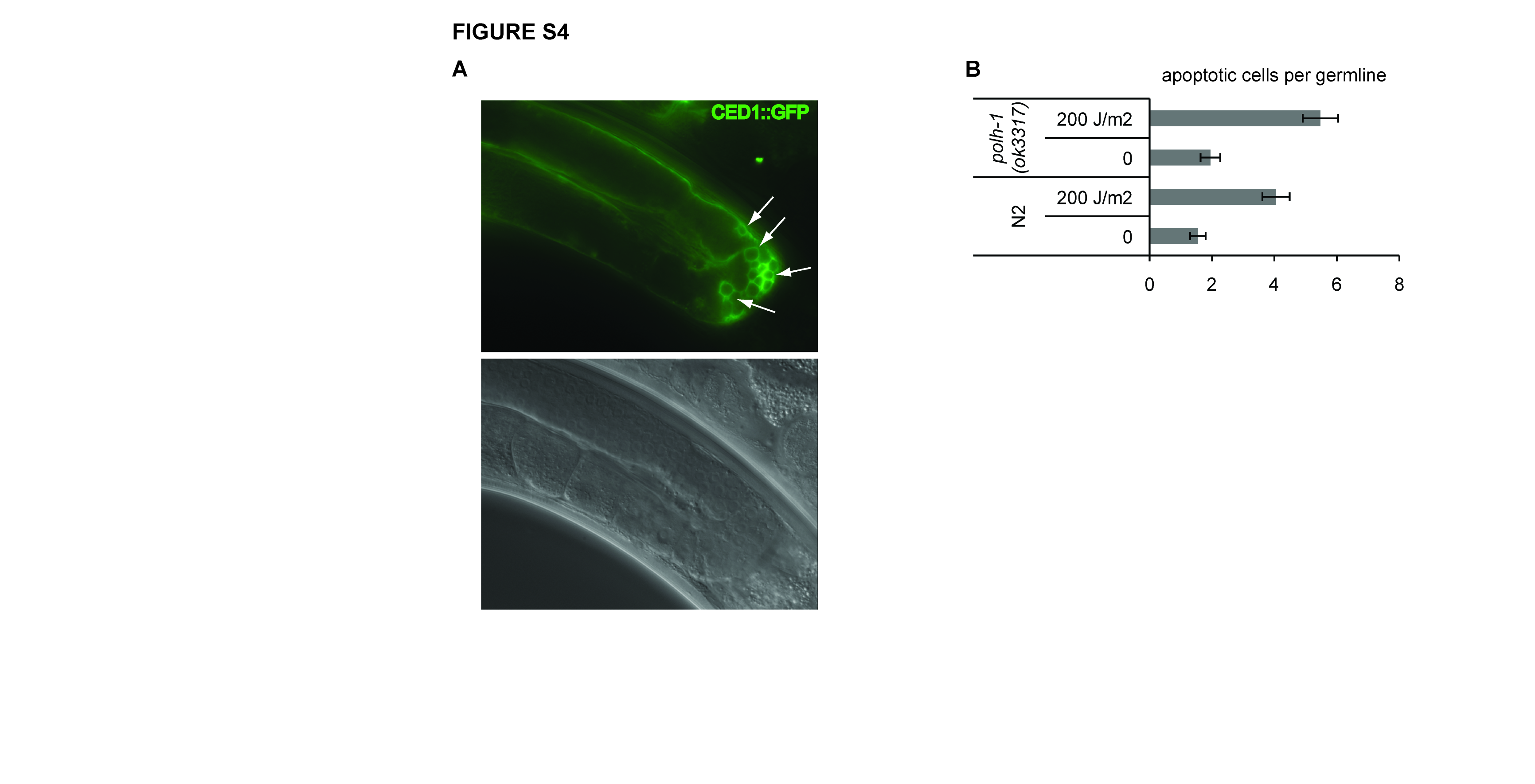

Supplement: Figure S4 — Apoptosis in the germline after UV-irradiation. (A) A transgenic line expressing pLim7::ced1::GFP is used. Examples of CED1-GFP engulfed cells in the germline bend are indicated with arrows. (B) Quantification of CED1-GFP engulfed cells. In the polh-1(ok3317) mutant apoptosis is slightly increased after exposure to UV irradiation. About 40 germlines have been analysed per sample. Error bars denote s.e.m. (TIF) [file pgen.1002800.s004.tif]

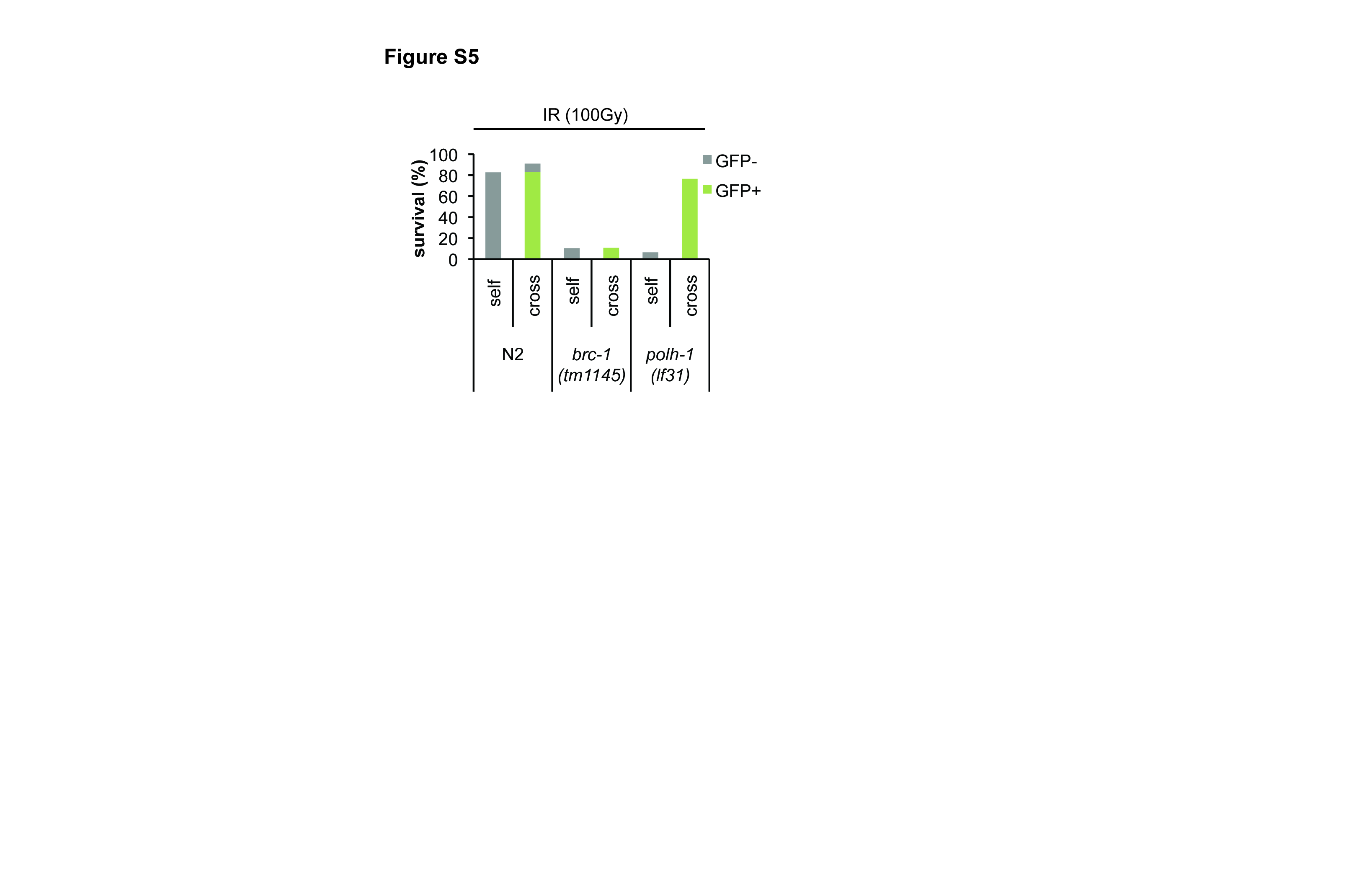

Supplement: Figure S5 — γ-irradiated hermaphrodites were crossed with non-irradiated males carrying a Pmyo-2::GFP transgene. Lethality induced by γ-irradiation is almost fully rescued in the cross progeny of polh-1, but not brc-1 hermaphrodites. (TIF) [file pgen.1002800.s005.tif]

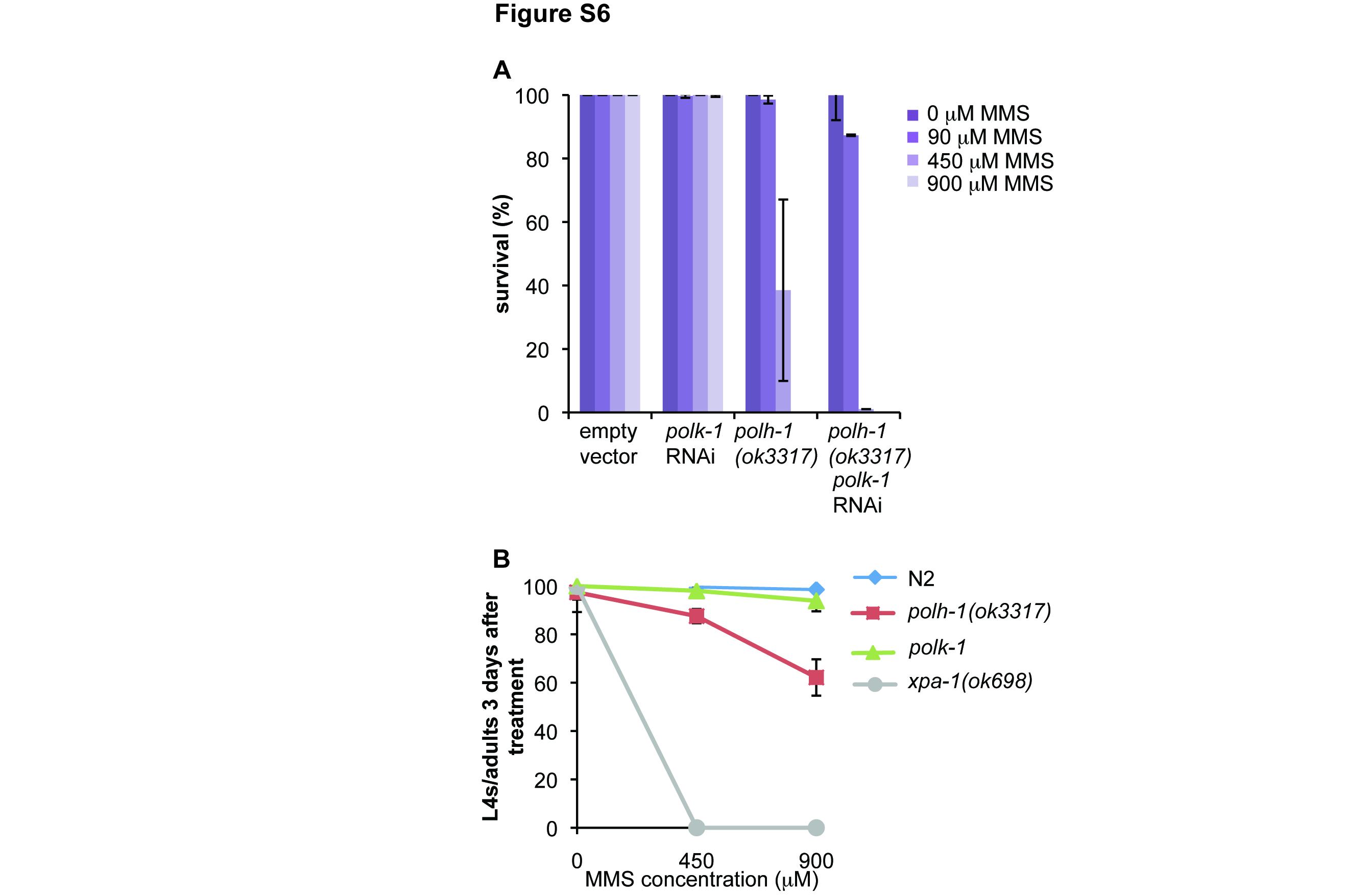

Supplement: Figure S6 — POLH-1 and POLK-1 in cellular tolerance to MMS during embryogenesis and L1 outgrowth. (A) MMS sensitivity of N2 and polh-1(ok3317) mutants with or without depletion of POLK-1 by RNAi. (B) Development of larvae exposed to MMS from L1 stage. Each line is the mean of three independent experiments; error bars denote s.e.m. (TIF) [file pgen.1002800.s006.tif]

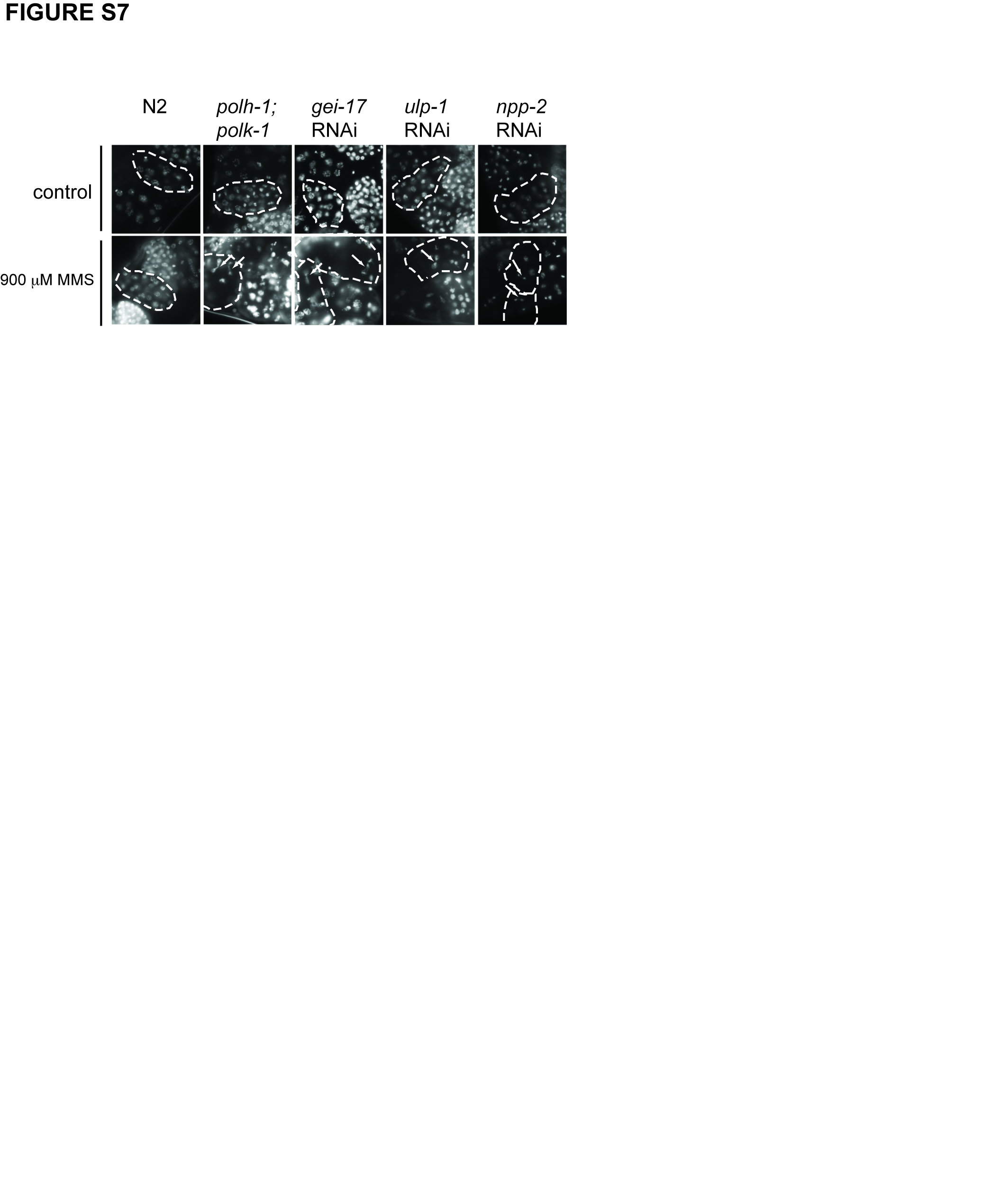

Supplement: Figure S7 — Morphological defects in MMS exposed embryos. DAPI-stainings of whole animals exposed for 24 hrs to MMS reveal a delay in development on indicated RNAi foods. Incidentally, chromatin bridges are visible (arrows) indicative of incomplete DNA replication. (TIF) [file pgen.1002800.s007.tif]
