# Supplementary material for: A Broad Requirement for TLS Polymerases η and κ, and Interacting Sumoylation and Nuclear Pore Proteins, in Lesion Bypass during C. elegans Embryogenesis
Source: PLoS Genet. 2012 Jun 28;8(6):e1002800. doi: 10.1371/journal.pgen.1002800 (PMC3386174; doi:10.1371/journal.pgen.1002800)
Supplement: Table S1 — List of newly generated strains used in this study. (DOC) [file pgen.1002800.s008.doc]

**Table S1**

| strain | genotype |
| --- | --- |
| XF110 | *polk-1(lf29)* |
| XF132 | *polh-1(lf31)* |
| XF656 | *polh-1(ok3317)* |
| XF242 | *polh-1(lf31);polk-1(lf29)* |
| XF507 | *polh-1(lf31);polk-1(lf29)* |
| XF729 | *polh-1(lf31); xpa-1(ok698)* |
| XF726 | *polh-1(ok3317);xpa-1(ok698)* |
| XF155 | *polh-1(lf31);brc-1(tm1145)* |
| XF153 | *polh-1(lf31);dog-1(gk10)* |
| XF504 | *polh-1(lf31);rde-3(ne298)* |
| XF496 | *brc-1(tm1145);rde-3(ne298)* |
| XF503 | *lfs129[P(elt-2)::GFP-HRreporter; rol-6(su1004);P(HS)::ISceI::Cherry]* |
| XF659 | *polh-1(ok3317); lfs129[P(elt-2)::GFP-HRreporter; rol-6(su1004);P(HS)::ISceI::Cherry]* |
| XF550 | *brc-1(tm1145); lfs129[P(elt-2)::GFP-HRreporter; rol-6(su1004);P(HS)::ISceI::Cherry]* |
| XF657 | *polh-1(lf31); bcIs39[P(lim-7)ced-1::GFP + lin-15(+)]* |
