# Supplementary material for: A Broad Requirement for TLS Polymerases η and κ, and Interacting Sumoylation and Nuclear Pore Proteins, in Lesion Bypass during C. elegans Embryogenesis
Source: PLoS Genet. 2012 Jun 28;8(6):e1002800. doi: 10.1371/journal.pgen.1002800 (PMC3386174; doi:10.1371/journal.pgen.1002800)
Supplement: Table S2 — polh-1 does not influence lethality in a mutant background where transposition is desilenced. Double mutants for the HR gene brc-1 and the mutator gene rde-3 display synthetic lethality while polh-1; rde-3 double mutants are comparable to rde-3 single mutants. (DOC) [file pgen.1002800.s009.doc]

**Table S2.** Survival of single and double mutants in transposition silencing.

| Genotype | Survival* |
| --- | --- |
| *rde-3 (ne298)* | 88 (±10) % |
| *rde-3(ne298); brc-1 (tm1145)* | 25 (±20) % |
| *rde-3 (ne298); polh-1 (lf31)* | 96 (±15) % |

* progeny of at least 500 individuals from minimal 12 worms has been counted
